# Supplementary material for: Polymeric Microspheres Designed to Carry Crystalline Drugs at Their Surface or Inside Cavities and Dimples
Source: Pharmaceutics. 2023 Aug 15;15(8):2146. doi: 10.3390/pharmaceutics15082146 (PMC10460081; doi:10.3390/pharmaceutics15082146)
Supplement: Supplementary file 1 [file pharmaceutics-15-02146-s001.zip › pharmaceutics-2441630-supplementary.pdf]

Supplementary Material to "Polymeric microspheres designed to carry crystalline drugs at their surface or inside cavities and dimples"
Part I

Figure S1.1 FTIR spectrum of the pristine microspheres of this study.

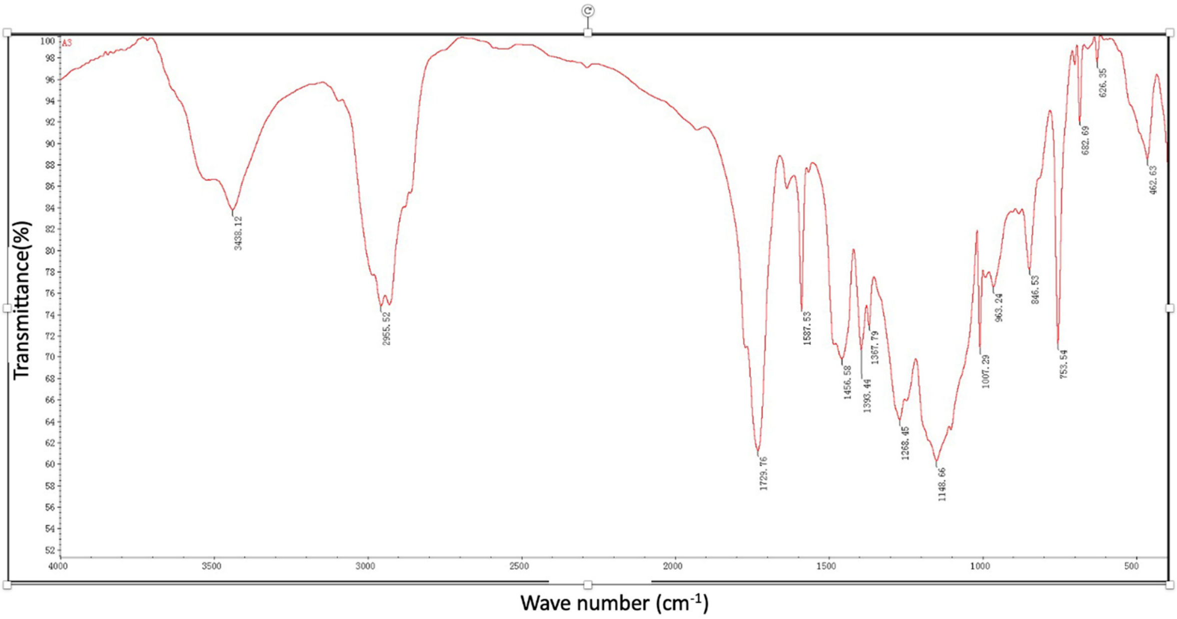

Part 2

Cytotoxicity in vitro of the unloaded microparticles.

Evaluation of the cytotoxicity of the pristine microspheres *in vitro* was conducted through experiments with human corneal epithelial cells (HCECs), which are known to be very sensitive and susceptible to toxic effects exerted by artificial materials. The HCECs were cultured in DMEM supplemented with 10 % FBS, penicillin (100 units/mL), and streptomycin (100 µg/mL). All cultures were maintained at 37 °C under 5 % CO_2 for 24 h. HCECs were seeded in a 24-well plate and divided into four groups: (i), control = no microparticles; (ii), 100-200 µm diameter range; (iii), 200-300 µm diameter range; (iv) 300-500 µm diameter range). Initial density was 20,000 cells/well. Cells were incubated for another 24 h. Subsequently, the medium was replaced with fresh DMEM, and the cells were incubated with 1 mg of different diameters of microspheres for 4 h. Then, the cells were rinsed with PBS buffer, and a fresh culture medium was added. After 24 h of incubation, 400 µL fresh culture medium with 40 µL CCK-8 solution were added into each well, followed by incubating at 37 °C for an additional 4 h. Finally, the absorbance at 450 nm was measured by a microplate

reader (SpectraMax i3x, Molecular Devices), and the cell viability was determined according to the following equation:

$$Cell\ viability\ (\%) = \frac{A_{450\ nm,sample} - A_{450\ nm,blank}}{A_{450\ nm,control} - A_{450\ nm,blank}} \times 100\%$$

Where $A_{450\ nm,\ sample}$ and $A_{450\ nm,\ control}$ are the absorbance in the presence and absence of microparticles, respectively. $A_{450\ nm,\ blank}$ is the absorbance of the well with 40 μL of CCK-8 without cells. All the samples were evaluated in six-fold (n = 6).

The results are shown schematically in the graph below. Cell viabilities were found to be higher than the control group (for the 2 groups of relatively small microspheres, i.e., 100-200 and 200-300 μm diameter range), or equal the control group (300-500 mm diameter range). These data indicate that the pristine microspheres are non-cytotoxic, which is completely in line with our previous work.

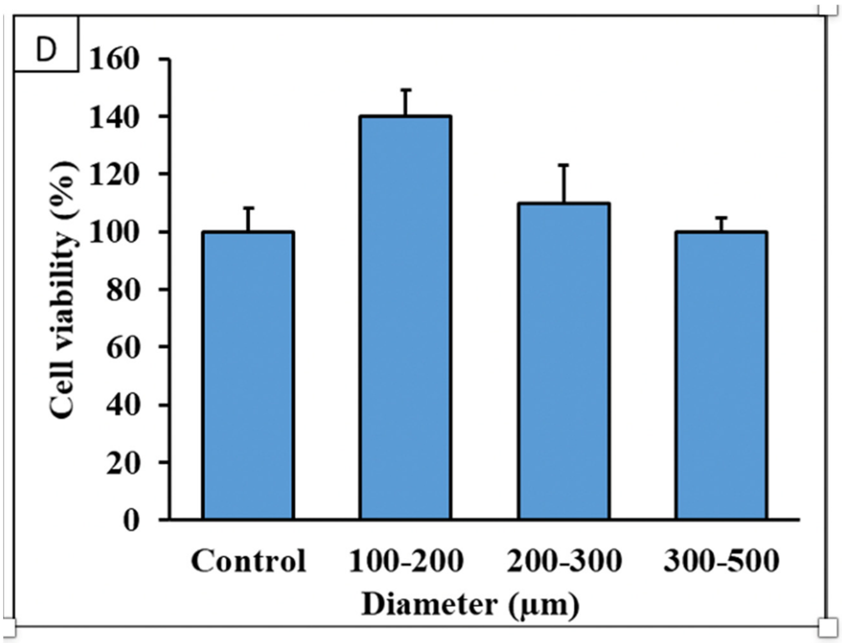

Figure S2.1

Biocompatibility of the pristine microspheres was studied further (again in vitro) by culturing cells in the presence of the particles. In these experiments, both human HCECs (as above) and L929 (mouse fibroblast cells) were used. Cells were observed by optical microscopy. The same amount of 50-80μm and 80-100μm microspheres were co-cultured with the HCECs

and L929 cells for 24 hours, and then placed under a light microscope for observation and photography. The results are shown below:

Photos taken by optical microscope

Control **50-80μm** **80-100μm**

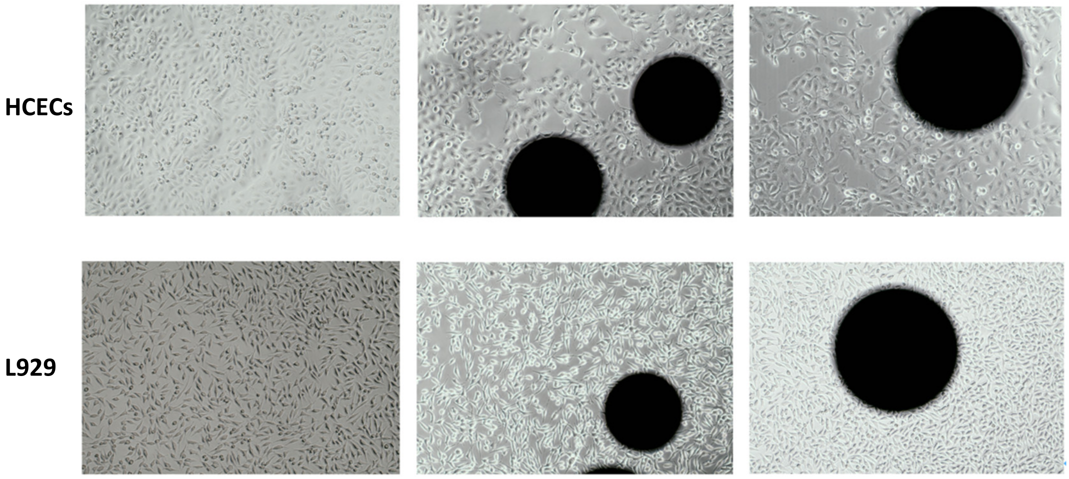

Figure S2.2

These images reveal that both cell types cells grow normally, also in the vicinity of the microspheres. No toxicity effects are seen, which is in line with the results of the CCK8 experiments.

Furthermore, HCECs and L929 cells were stained with Calcein-AM and propidium iodide (PI). Under the fluorescence microscope, live cells appear green and dead cells appear red. After the different sizes of microspheres were co-cultured with HCECs and L929 cells for 24 hours, most of the HCECs and L929 cells in each group were living green cells in good growth condition. Few dead cells (red) were encountered; see images below. The results were consistent with the CCK-8 cytotoxicity results. The results of live and dead cell staining showed that the microspheres had good biocompatibility and did not change the morphology and relative viability of HCECs and L929 cells, which was suitable for further biomedical applications.

HCECs Live Death Staining

Control **50-80μm** **80-100μm**

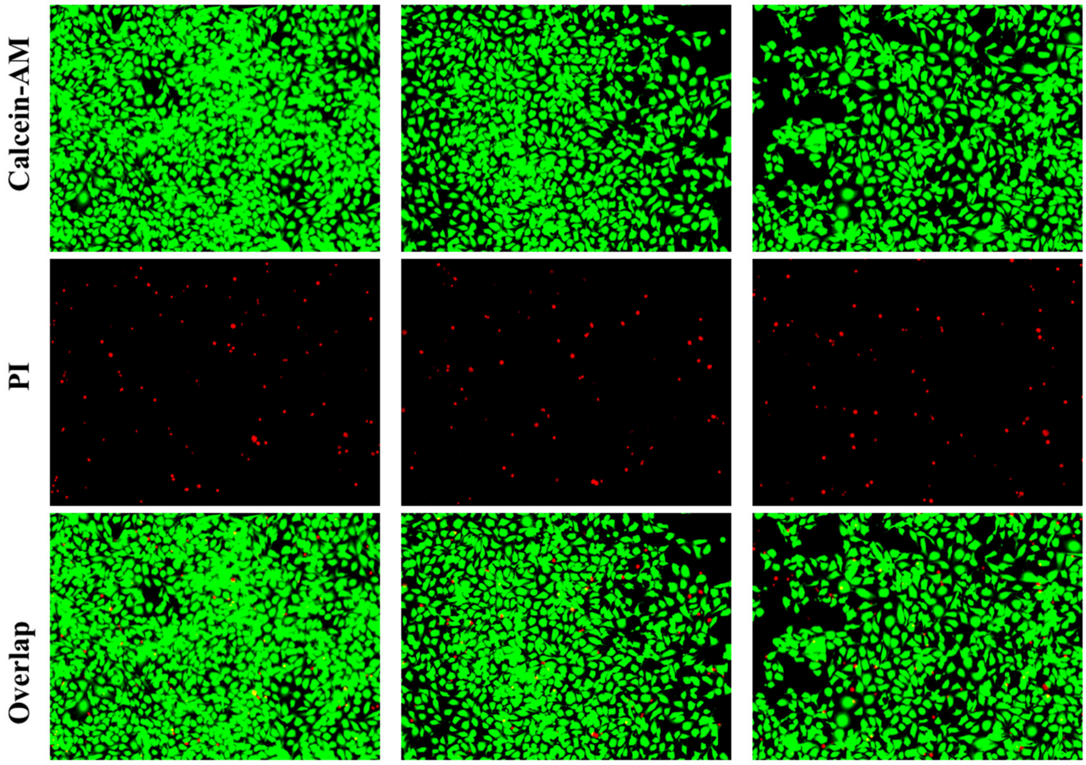

Figure S2.3 Cell death/viability staining to observe the growth status of HCECs

L929 Live Death Staining		
Control	50-80μm	80-100μm

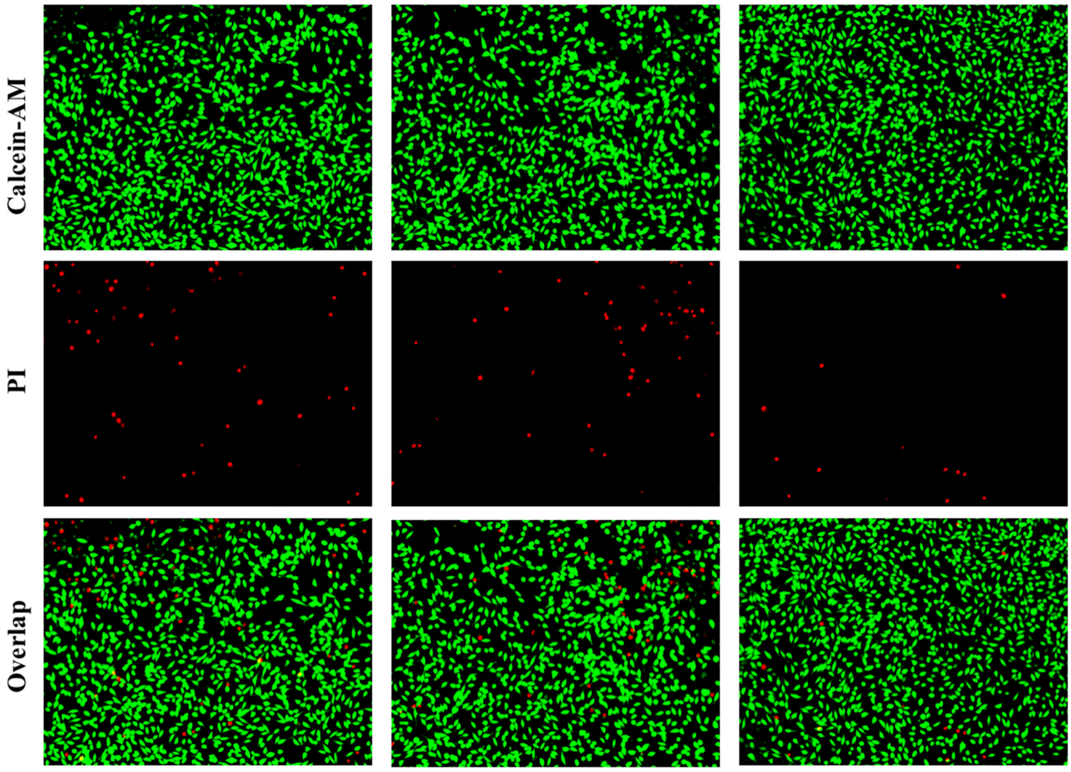

Figure S2.4: Cell death/viability staining to observe the growth status of L929
